# Efficient Isolation of Mycosporine-Like Amino Acids from Marine Red Algae by Fast Centrifugal Partition Chromatography

**DOI:** 10.3390/md20020106

**Published:** 2022-01-27

**Authors:** Michael Zwerger, Stefan Schwaiger, Markus Ganzera

**Affiliations:** Department of Pharmacognosy, Institute of Pharmacy, University of Innsbruck, A-6020 Innsbruck, Austria; Michael.J.Zwerger@uibk.ac.at (M.Z.); Stefan.Schwaiger@uibk.ac.at (S.S.)

**Keywords:** mycosporine-like amino acids, MAA, isolation, FCPC, shinorine, porphyra-334

## Abstract

Marine rhodophyta are known to synthesize specific secondary metabolites, mycosporine-like amino acids (MAAs), to protect themselves from harmful UV-radiation. Shinorine and porphyra-334 are among the most abundant representatives of this compound class. In the present work, a novel approach for their isolation is described. As a first step, a fast centrifugal partition chromatography method, with an aqueous two-phase system comprising water, ethanol, ammonium sulfate and methanol in ascending mode, was developed to isolate the two MAAs from crude aqueous-methanolic extracts of three algal species within 90 min. The compounds could be isolated when just one of them was present in a sample or also both at the same time. By employing solid phase extraction as a second purification step, the individual MAAs were obtained in high purity and good quantity within a much shorter time frame than the established purification protocols, e.g., semi-preparative HPLC. For example, from 4 g *Porphyra* sp. (Nori) crude extract, 15.7 mg shinorine and 36.2 mg porphyra-334 were isolated. Both were highly pure, as confirmed by TLC, HPLC-MS and NMR analyses.

## 1. Introduction

Many marine organisms have developed specific strategies to protect themselves from harmful UV-A and UV-B radiation. One is to synthesize unique secondary metabolites like MAAs [1,2]. Structurally, they consist of a core cyclohexenone or cyclohexenimine ring, substituted with amino alcohols or amino acids. They are nitrogen rich, have low molecular weight (<400 Da) and are water-soluble compounds with high polarity [1,3,4,5]. In macroalgae, the most abundant representatives are shinorine, porphyra-334 and palythine [3]. Due to their extremely strong absorption, from 310 to 360 nm, they are amongst the most efficient natural sunscreens [1,5], and commercial products like Helioguard^®^ 365, a formulation comprising porphyra-334 and shinorine developed by Schmid et al. [6], are available already. Promising effects on immunostimulation, DNA protection, collagenase inhibition and wound healing have been reported for MAAs as well [5].

The interest in MAAs is steadily growing. However, use and in-depth characterization are hindered by their elaborate isolation and thus limited supply. Current purification strategies include the use of semi-preparative HPLC, combined with either column chromatography (normal and reversed-type materials) [1] or solid phase extraction [7] as preceding steps. Other protocols are solely based on HPLC in analytical scale [3] or several LC-columns in series [4]. All of them are tedious and time consuming, as the isolation of low abundant, highly polar and structurally similar compounds is always challenging. Furthermore, purity of the isolated MAAs is often only confirmed by UV spectroscopy or HPLC using a diode array detector (DAD). This is inadequate, as possible impurities like amino acids or sugars cannot be detected. Furthermore, for correct structural identification, purity is a crucial factor for all further studies, including biological testing. Thus, it needs to be assured by a combination of complementary techniques like TLC, HPLC-mass spectroscopy (MS) and nuclear magnetic resonance (NMR) spectroscopy.

Fast centrifugal partition chromatography (FCPC) is a variant of countercurrent chromatography (CCC), with a history of use dating back to the 1980s [8]. CCC comprises all forms of liquid–liquid chromatography, where analytes are separated due to their different partition in an immiscible biphasic fluid system. Accordingly, there is no solid stationary phase as in conventional chromatography, but rather a liquid that is held inside the device by centrifugal forces while the mobile phase (also a liquid) passes through. As a consequence, no loss of the sample through irreversible binding to the stationary phase can occur. FCPC methods can easily be upscaled by changing the rotor size. Additionally, they are fast, flexible in terms of analytes and fluid systems, and cost saving [9,10]. This renders FCPC ideal for the purification of natural products in complex matrices [10,11].

Natural products of diverse polarity have successfully been isolated by FCPC in the past, including flavonoids, alkaloids, anthraquinones, terpenoids and saponins [11,12,13]. However, conventional two-phase systems do not permit the separation of highly polar compounds, because they will not distribute between the phases as required [8,14]. This led to the development of aqueous two-phase systems (ATPS) which, for example, enabled the separation of polysaccharides or the precipitation of proteins in biological samples [15,16]. In ATPS, two immiscible phases are created by adding inorganic salts or polymers like polyethylene glycols to water as the main constituent. Respective systems are described to offer mild and stable separation conditions, combined with high separation efficiency [9]. However, they have never been evaluated for the purification of MAAs.

The present work describes the development of an FCPC based isolation protocol for shinorine and porphyra-334 from the crude extracts of three marine algae, namely *Gracilaria gracilis, Spongoclonium pastorale* and *Porphyra* sp. The latter is also known as Nori and is primarily used in Asian cuisine. This new approach should not only surpass the currently used ones in terms of speed and efficiency, but also result in compounds with high purity and yield.

## 2. Results and Discussion

### 2.1. Selection of Solvent Systems

The first step in the development of any FCPC method is the selection of an optimal two-phase solvent system. It should exhibit different partition coefficients (*K*) for the desired compounds in the range of the so-called sweet spot between 0.4 and 2.5, as well as a separation factor *α* over 1.5 (*α* = *K*_2_/*K*_1_). This would permit elution of the target compounds and avoid coelutions [14,17]. However, not only the *K* values of the analytes are relevant; so are those of other matrix compounds which could interfere. To account for these issues, close to 40 different solvent combinations were evaluated for their suitability to isolate MAAs by FCPC (see Table 1 and Supplementary Information, Table S1 for the tested systems).

Among the options tried were the commonly used HEMWat (*n*-hexane/ethyl acetate/methanol/water) and ChMWat (chloroform/methanol/water) systems. However, due to their polarity, both MAAs were only found in the water phase. Thus, biphasic systems for more polar analytes were considered, but neither EBuWat (ethyl acetate/*n*-butanol/water) nor terAcWat (MTBE/acetonitrile/water) systems showed improved results. Again, the target compounds remained solely in the aqueous layer, so that it became evident that both phases had to be extremely polar. A similar observation was made concerning the separation of catecholamines by FCPC [8,9], so that their described ATPS were considered for the current application. At first, systems comprising variable amounts of *n*-butanol, ethanol, saturated ammonium sulfate solution and water were evaluated. It was noticed that *n*-butanol was less favorable for the separation of MAAs and it was therefore replaced with a small percentage of methanol. Furthermore, the salt content was reduced without any negative effects on the partition of MAAs. The finally selected system was composed of 51.4 w% water, 28.0 w% ethanol (96 v%), 18.2 w% ammonium sulfate and 2.4 w% methanol, resulting in a settling time of 33 s. A further increase of the methanol content was not possible as this resulted in salt precipitation. Under these conditions, porphyra-334 and shinorine had *K* values of 1.31 and 0.81, respectively, as well as an α value of 1.61, which indicated that their separation by FCPC should be possible (Table 1). An ATPS containing a low-density polymer (PEG 400) also enabled a separation of the two MAAs, but the calculated *α* value was inferior and the general handling (high viscosity of the polymer containing phase) unfavorable, so this option was not pursued.

The same salt-based ATPS was used for sample preparation. Besides the good solvating power for MAAs, an additional positive effect was observed; when mixing the crude extracts with this biphasic system, a dense, gelatinous middle layer formed. It contained no MAAs (confirmed by HPLC, data not shown) but possibly proteins, because ATPS systems are also used for protein precipitation [18,19]. Therefore, a pre-purification of the sample was achieved without the necessity of an additional treatment step.

### 2.2. Initial FCPC Experiments

FCPC separations were carried out in ascending mode, meaning the upper phase of the selected system acted as the mobile phase and the lower one as the stationary phase, respectively. This was advantageous because of the lower salt content in the upper phase, which was subsequently easier to remove from the collected fractions.

On the instrument with a small 55 mL rotor, the influence of different FCPC parameters was evaluated first, using two species which contained one MAA each (porphyra-334 in *Spongoclonium pastorale* and shinorine in *Gracilaria gracilis*). It was observed that an increase in elution speed up to 1.25 mL/min was advantageous in terms of peak shape and analysis time for the purification of MAAs. In order to compensate for a loss of more than 50% of stationary phase at this flow rate, the rotation speed was increased to 900 rpm, a good compromise between stationary phase retention (S_f_ was 65%) and stable performance of the instrument. Experiments were conducted with 50 mg and 100 mg of crude extract (dissolved in a total of 3 mL of the biphasic system). Even with the higher amount, no deteriorated peak shapes were observed.

### 2.3. Isolation of Single MAAs by FCPC

**Figure 1:**
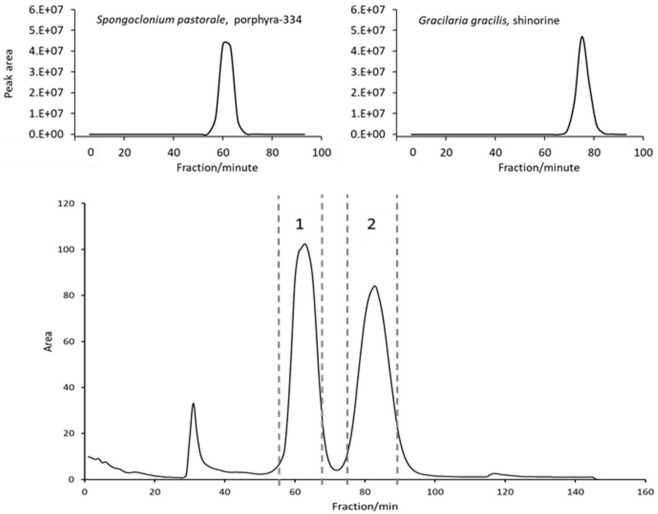
FCPC separation of the crude extracts of three algae using the developed ATPS system (51.4 w% water, 28.0 w% ethanol (96 v%), 18.2 w% ammonium sulfate, 2.4 w% methanol) in ascending mode. Above: reconstructed chromatograms of *G. gracilis* and *S. pastorale* based on HPLC-analysis of individual fractions at 330 nm according to literature [22]; Below: FCPC online chromatogram of *Porphyra* sp. separation (2 g extract), dashed lines indicate pooled fractions.

**Figure 2:**
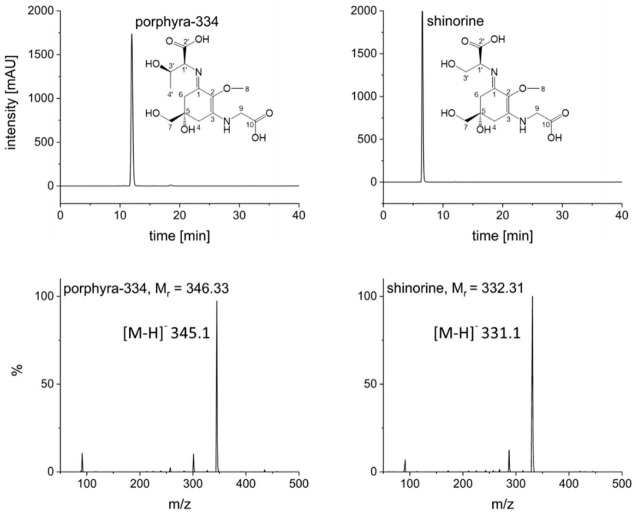
HPLC analyses of pure compounds at 330 nm and corresponding mass spectra; HPLC conditions according to [22], MS conditions: ESI negative mode, capillary voltage: 4500 V, drying gas (nitrogen): 12 L/min at 320 °C, nebulizer gas: 1.73 bar, scan range: 50 to 500 *m/z*.

Naturally, to scale up to the 1 L rotor, some parameters had to be adjusted. Flow rate was increased to 20 mL/min and rotation speed set to 1300 rpm in order to ensure a comparable stationary phase retention (S_f_ = 63%); these settings resulted in a stable backpressure of 60 bar. Two grams of crude extract could be injected per run (see experimental section for sample preparation). The FCPC separation of *S. pastorale* and *G. gracilis* extracts revealed one symmetric peak each, corresponding to the contained MAA (Figure 1). As the online recorded chromatograms showed an unstable baseline—especially at the beginning, possibly due to minor stationary phase extrusion and therefore opaque appearance of the eluate interfering with the detector (see Supplementary Information, Figure S3)—individual fractions were analyzed by HPLC, and the chromatograms reconstructed based on the recorded peak area at 330 nm. Furthermore, it proved to be necessary to monitor separation efficiency and compound purity by complementary techniques. For this and all fractionations to follow, the possible coelution of other compounds was monitored by TLC instead of LC-MS, because of the high number of fractions and the incompatibility of MS with the high salt concentration in the samples. For example, when isolating shinorine from *G. gracilis*, TLC results indicated a second compound (most likely a sugar, not visible in UV but only after spraying with anisaldehyde/sulphuric acid reagent) eluting right after the desired MAA. It could be removed by correct fractionation (Supplementary Information, Figure S6). Additionally, intensively colored constituents always eluted before the target compounds, but they could be excluded easily. Overall, this resulted in one MAA-containing fraction per algae of more than 3 g (mainly salt). Removing the salt by dialysis, which is common for large biomolecules like polysaccharides [20], was not possible, as the MAAs were too small in size. Hence, due to the insolubility of ammonium sulfate in short-chain alcohols [21], the fractions were extracted five times with 10 mL cold and water-free methanol by sonication for 15 min each. After centrifugation (5 min, 1500× *g*) the supernatants were combined, evaporated to dryness and lyophilized. This resulted in shinorine enriched fractions of 16.4 mg (*G. gracilis*) and 52.2 mg (*S*. *pastorale*), which were finally purified by SPE following a well-established procedure [7]. The selected mixed mode phase (Oasis MCX) removed any possible remaining traces of salt as well as other impurities in a timely manner. As determined by HPLC analysis, this step resulted in a minor loss of MAAs; 87.8% of the applied shinorine and 85.9% of porphyra-334 could be recovered based on the overall amount of MAAs applied on the SPE cartridge. The obtained pure MAAs were lyophilized and subsequently analyzed by HPLC, HPLC-MS, TLC and NMR to confirm purity and identity. For MS data, see Figure 2. The HPLC-chromatograms at 210 nm (Figure S2), original ^1^H-NMR spectra (Figures S9 and S10) and NMR shift values in comparison to literature (Table S2) are shown as Supplementary Information. All results were in good agreement with published data and indicated pure compounds. The final yields, always related to 2 g of crude extract, were 5.7 mg shinorine from *G. gracilis* and 7.5 mg porphyra-334 from *S. pastorale*. Close to 60% of the originally present MAAs (as determined by HPLC according to [22]), i.e., 59.6% of shinorine and 57.2% of porphyra-334, could be recovered after the entire purification procedure.

### 2.4. Isolation of MAAs from Porphyra sp.

Both above mentioned species contained a single MAA. It was therefore of interest to evaluate whether both could be separated at the same time using FCPC. We selected a commercially available Nori sample (*Porphyra* sp.) for our study, as both desired MAAs were present in high concentrations. Indeed, the FCPC chromatogram suggested a successful separation by showing two symmetric, baseline resolved peaks (Figure 1). Notably, only 90 min were required for this step. Fractions 58–69 contained porphyra-334 and fractions 76–90 shinorine, which agreed with the separations described before. Additionally, the recovery rates after SPE purification (84.6% for porphyra-334 and 90.2% for shinorine), calculated in reference to the total amount of MAA on the column, were similar. TLC analysis again showed the (successful) removal of a sugar, yet spraying the plate with ninhydrin reagent after SPE purification indicated a further compound in the shinorine fraction. This impurity, possibly a small peptide or amino acid, could be removed by Sephadex LH-20 column chromatography (550 × 10 mm) using water as mobile phase and a pump delivering a steady flow of 1.0 mL/min. This approach was definitely not as efficient as semi-preparative HPLC. However, larger amounts of purified compounds could be obtained in a much faster and more economical way, and it can be easily scaled up. Shinorine eluted after approx. 25 min and could be resolved from the impurity by appropriate fractionation. However, this step resulted in a loss of 44% of the MAA. This might seem like a high number, but the obtained substance was highly pure, as determined by TLC, LC-MS and NMR. Furthermore, this interfering compound was not observed during the isolation of shinorine from *G. gracilis*, so this additional purification step will only be required in selected cases.

As a final proof of concept, the entire purification procedure was repeated with 4 g instead of 2 g Nori extract. Both MAAs could be isolated with the same purity, confirming the reproducibility of the method and indicating its potential for even further upscaling. At the end, 15.7 mg shinorine (0.39% of the extract) and 36.2 mg porphyra-334 (0.91%) were obtained after FCPC, SPE and, if required, Sephadex LH-20 column chromatography, equivalent to a total MAA yield of 33.2% and 56.7%, respectively.

## 3. Materials and Methods

### 3.1. Plant Material and Preparation of Crude Extracts

Nori (*Porphyra* sp.) was obtained from a local supermarket in Innsbruck 2021. *Gracilaria gracilis* was collected in 2018 in Brittany (France) and morphologically identified by Prof. U. Karsten from the University of Rostock, Germany. *Spongoclonium pastorale* was collected and identified as described previously [1]. Voucher specimens of all samples were deposited at the Department of Pharmacognosy, University of Innsbruck, Austria. The dried algal material was finely powdered and extracted according to literature, i.e., four times with 20% methanol in water in an ultrasonic bath (Bandelin Sonorex, Berlin, Germany) for 15 min at ambient temperature [23]. The solution was centrifuged (10 min at 1500× *g*), the clear supernatants combined, and the solvents removed under vacuum. To ensure complete dryness, the extracts were subsequently lyophilized (Virtis bench top Pro, SP Scientific, Gardiner, NY, USA).

### 3.2. Chemicals and Reagents

All solvents and chemicals used in this study were purchased from Merck (Darmstadt, Germany). HPLC-grade water was obtained from an Arius purification system (Sartorius, Göttingen, Germany). Deuterated solvent for NMR experiments came from Euriso-Top (Saint-Aubin Cedex, France). Sephadex LH-20 material was purchased from GE Healthcare (Uppsala, Sweden).

### 3.3. Analytical Conditions

HPLC analyses were performed on a Merck–Hitachi Elite La Chrom instrument (Tokyo, Japan). Separation conditions were according to the method described by Orfanoudaki et al. [22], selecting 210 and 330 nm for detection. All samples were membrane filtered (0.45 µm pore size) prior to analysis. NMR experiments were conducted on a Bruker Avance III 400 HD spectrometer (Karlsruhe, Germany) operated at 400.13 MHz (^1^H-NMR). Mass spectra were recorded on an Agilent InfinityLab LC/MSD system comprising an Agilent 1260 HPLC (Santa Clara, CA, USA) coupled to a single quadrupole MS detector. The experiments were performed in negative ESI mode, using the following settings: capillary voltage 4500 V, drying gas (nitrogen) flow 12 L/min at 320 °C, nebulizer gas (nitrogen) 1.73 bar, and scan range from 50 to 500 *m/z*.

The purity of isolated compounds was also confirmed by TLC, using silica gel plates from Macherey–Nagel (Düren, Germany) and the solvent system developed by Hartmann et al., i.e., a mixture of n-butanol, acetic acid and water in the ratio 6:2:2 [7]. Two spray reagents were used for visualization, anisaldehyde/sulphuric acid (universal spray reagent) and ninhydrin (specific for peptides and amino acids). In both cases, the plates were heated to 100 °C for 5 min after spraying and then evaluated in the visible range by naked eye as well as at 366 nm.

### 3.4. Determination of Partition Coefficients (K)

*K* values were determined using the shaking flask method in a way that 600 µL each of upper and lower phase of a pre-equilibrated two-phase system were added to 2 mg of crude extract in an HPLC-vial. After vortexing for two minutes and phase separation, an aliquot of 300 µL of each phase was removed and dried under a stream of air. The residue was dissolved in 1 mL of water and analyzed by HPLC. *K* values were calculated as the ratio of MAA peak areas in the upper and lower phase, as determined at 330 nm.

### 3.5. FCPC Experiments

For initial screening, an FCPC instrument from Kromaton (Annonay, France) with a 55 mL rotor was used. The latter consisted of 15 discs with 60 twin cells each. Sample loop size was 5 mL, rotation speed adjustable up to 3000 rpm, and the solvent delivered by a Merck–Hitachi L-7100 pump (flow rate 1.25 mL/min). All final purifications were performed on a Gilson CPC 1000 instrument (Middleton, WI, USA) with a rotor capacity of 1 L. Rotation speed was set to 1300 rpm. The instrument was coupled to a Gilson-PLC unit composed of a quaternary pump (flow rate 20 mL/min), 50 mL sample loop and a DAD set to 330 nm; fractionation time was 1 min. Every third tube was additionally analyzed by HPLC, i.e., the solvent was evaporated, the residue extracted with MeOH (to remove salt), the sample dried again and dissolved in water prior to analysis.

Best results were obtained with an ATPS comprising 51.4 w% water, 28.0 w% ethanol (96 v%), 18.2 w% ammonium sulfate and 2.4 w% methanol. After weighing all reagents, ammonium sulfate was dissolved in water by sonication, and then ethanol and methanol were added. The two-phase system was transferred to a separatory funnel and vigorously equilibrated by shaking for several minutes. After the upper and lower phases partitioned, they were separated and degassed by sonication right before use. The upper phase was used as mobile phase, whereas the salt-rich lower phase served as stationary phase (ascending mode). Prior to starting a run, the system was filled with stationary phase and equilibrated with mobile phase at the selected rotation speed and optimal flow rate. Stationary phase retention volumes (S_f_) were monitored by collecting the effluent during the equilibration phase and measuring the displaced volume of stationary phase.

When using the 1 L rotor, the sample (2 or 4 g of crude extract) was extracted twice with a 1:1 mixture of upper and lower phase (15 mL per extraction) by sonication for 5 min and then centrifuged (5 min, 1500× *g*). The solvents were decanted and injected (both phases) into the loop. With the small rotor the same procedure was applied, yet using only 1.5 mL per extraction step (totally injected volume 3 mL).

### 3.6. SPE

SPE purification of the compounds was achieved using Oasis MCX cation exchange cartridges from Waters (Milford, MA, USA), following the slightly modified protocol of Hartmann et al. [7]. The cartridges were conditioned with one column volume of methanol and water each, before applying the aqueous sample solution in a concentration that did not exceed 10% of the sorbent mass. After washing the columns with two volumes of water, elution of the MAAs was achieved by flushing with two volumes of 5% acetic acid in water.

## 4. Conclusions

The potential of MAAs like shinorine and porphyra-334 to absorb harmful UV radiation in a very efficient way has evoked interest in this compound class, including commercial applications. This highlighted the need for faster and more economic methods of separation and purification on larger scales—because currently available approaches are very time consuming.

Within this study, an isolation protocol for two abundant MAAs employing FCPC was developed, and successfully applied to purify shinorine and porphyra-334 from the crude extracts of different algae, including one species (*Porphyra* sp.) that is commercially available in large quantities. *Gracilaria gracilis* and *Spongoclonium pastorale* have never been used for the isolation of MAAs before. The separation of two compounds might not seem a difficult task at first glance. However, their very high polarity, close structural resemblance, low abundance, and extremely high UV absorption, possibly masking other constituents, rendered this attempt a very challenging one. Additionally, impurities like sugars or amino acids with no UV absorbance were considered. The presented technique impressed with significant advantages over previous procedures. First, the overall required time could be considerably shortened (e.g., one FCPC run required only 90 min). Second, basically no toxic organic solvents were required (e.g., this was a sustainable approach). Third, the method has potential for upscale. All these aspects are prerequisites for further use and biological characterization of MAAs. The approach described herein will be very helpful in this respect.

## Figures and Tables

**Table 1:** Selection of biphasic systems evaluated for the purification of MAAs.

Solvent System	Ratio (*v*/*v*) if Not Stated Otherwise	K_ascending_ Shinorine	K_ascending_ Porphyra-334	α
ethyl acetate/n-butanol/water	6:4:10	∞	∞	
ethyl acetate/n-butanol/water	0:10:10	∞	∞	
n-hexane/EtOAc/MeOH/water	3:7:3:7	∞	∞	
n-hexane/EtOAc/MeOH/water	1:9:1:9	∞	∞	
chloroform/MeOH/water	10:5:5	0	0	
chloroform/MeOH/water	10:7:3	0	0	
EtOAc/MeOH/water	6:1:3	∞	∞	
n-butanol/acetic acid/water	4.4:0.6:5	∞	∞	
n-butanol/MeOH/water	4:1:5	25.70	17.92	
PEG 400/sodium sulfate/water	20%/16%/64% (*w*/*w*)	0.88	0.67	1.33
n-butanol/EtOH 96v%/saturated ammonium sulfate solution/water	1.75:0.125:1:1	∞	∞	
n-butanol/EtOH 96v%/saturated ammonium sulfate solution/water	0.5:0.75:1:1	13.61	7.82	
EtOH 96v%/ammonium sulfate/water	28.1%:20.3%:51.6% (*w*/*w*)	1.52	0.96	1.58
EtOH 96v%/ammonium sulfate/water/MeOH	28.0%/18.2%/51.4%/2.4% (*w*/*w*)	1.31	0.81	1.62

*K* values, partition coefficient, *α*, separation factor, ∞, all relevant analytes in lower phase, 0, all relevant analytes in upper phase.

## Supplementary Materials

The following supporting information can be downloaded at: https://www.mdpi.com/article/10.3390/md20020106/s1, Figure S1: HPLC analysis of the crude algal extracts; Figure S2: HPLC analysis of pure shinorine and porphyra-334; Figure S3: Online chromatograms recorded during the FCPC separation of *G. gracilis* and *S. pastorale*; Figure S4: Removal of an impurity by fractionation exemplarily shown for *Porphyra* sp.; Figure S5: Purification of shinorine by Sephadex LH-20 column chromatography; Figure S6: TLC of the pure compounds isolated from algae containing only one MAA; Figure S7: TLC of the pure MAAs isolated from *Porphyra* sp. (Nori); Figure S8: Total Ion Current (TIC) chromatogram of pure shinorine and porphyra-334; Figure S9: ^1^H NMR spectrum of porphyra-334 (isolated from *Porphyra* sp.); Figure S10: ^1^H NMR spectrum of shinorine (isolated from *Prophyra* sp.); Table S1: Additionally evaluated FCPC-systems; Table S2: ^1^H-NMR data of isolated compounds in comparison to literature values.
