# Supplementary material for: Efficient Isolation of Mycosporine-Like Amino Acids from Marine Red Algae by Fast Centrifugal Partition Chromatography
Source: Mar Drugs. 2022 Jan 27;20(2):106. doi: 10.3390/md20020106 (PMC8879178; doi:10.3390/md20020106)
Supplement: Supplementary file 1 [file marinedrugs-20-00106-s001.zip › marinedrugs-1571204-supplementary.pdf]

# Efficient isolation of mycosporine like amino acids from marine red algae by fast centrifugal partition chromatography

Supplementary information

Michael Zwerger, Stefan Schwaiger, Markus Ganzera*

Institute of Pharmacy, Pharmacognosy
University of Innsbruck
Innsbruck, Austria

*Corresponding author:
Assoz. Prof. Dr. Markus Ganzera
Institute of Pharmacy, Pharmacognosy, University of Innsbruck
Innrain 80-82, 6020 Innsbruck, Austria

# 1. Additionally evaluated FCPC-systems

Table S1. Additionally evaluated FCPC-systems

| Solvent system | Ratio ((v/v) if not given otherwise) | *K* shinorine | *K* porphyra-334 | *α* |
|---|---|---|---|---|
| **ethyl acetate/*n*-butanol/water** | 4:6:10 | ∞ | ∞ | |
| **ethyl acetate/n-butanol/water** | 2:8:10 | ∞ | ∞ | |
| **MTBE/ACN/water** | 4:6:10 | ∞ | ∞ | |
| ***n*-hexane/ethyl acetate/MeOH/water** | 2:8:2:8 | ∞ | ∞ | |
| ***n*-hexane/ethyl acetate/MeOH/water** | 0:10:0:10 | ∞ | ∞ | |
| **chloroform/MeOH/water** | 10:6:4 | 0 | 0 | |
| ***n*-butanol/EtOH/water** | 4:1:4 | ∞ | ∞ | |
| **toluol/formic acid/water** | 4:1:5 | ∞ | ∞ | |
| **1,2-dichloroethane/formic acid/water** | 4:2:4 | 0 | 0 | |
| **cyclohexane/acetic acid/water** | 4:3:3 | ∞ | ∞ | |
| ***n*-hexane/acetic acid/water** | 4:3:3 | ∞ | ∞ | |
| **toluol/acetic acid/water** | 4:3:3 | ∞ | ∞ | |
| **chloroform/formic acid/water** | 4:2:4 | 0 | 0 | |
| **diethylether/acetic acid/water** | 5:1:4 | ∞ | ∞ | |
| **chloroform/acetic acid/water** | 4:2:4 | 0 | 0 | |
| **1,2-dichloroethane/acetic acid/water** | 4:2:4 | 0 | 0 | |
| **isoamyl alcohol/acetic acid/water** | 5:1:4 | ∞ | ∞ | |
| **ethyl acetate/acetic acid/water** | 5:1:4 | ∞ | ∞ | |
| **ethyl acetate/n-butanol/water** | 2:4:4 | ∞ | ∞ | |
| **MTBE/1,2-dimethoxyethane/water** | 1:2:1 | ∞ | ∞ | |
| **isoamyl alcohol/MTBE/ACN/water+dodecylamine** | 3:5:1:7 + 0.8 g | ∞ | ∞ | |
| **isopropanol/saturated ammonium sulfate solution/water** | 1:1:1 | 6.09 | 3.85 | 1.58 |
| **EtOH 96 v%/ammonium sulfate/water/isopropanol** | 28.0%/18.2%/51.4%/2.4% (w/w) | 1.07 | 0.68 | 1.58 |
| **EtOH 96 v%/ammonium sulfate/water/MeOH +acetic acid (acidic condition)** | 28.0%/18.2%/51.4%/2.4% (w/w) | 1.11 | 0.70 | 1.59 |
| **EtOH 96 v%/ammonium sulfate/water/MeOH +ammonia (basic condition)** | 28.0%/18.2%/51.4%/2.4% (w/w) | 0.92 | 0.62 | 1.48 |

*K* …. partition coefficient
*α* ….. separation factor
0 …. all of the analyte in the upper phase
∞ …. all of the analyte in the lower phase

## 2. Selected HPLC Chromatograms

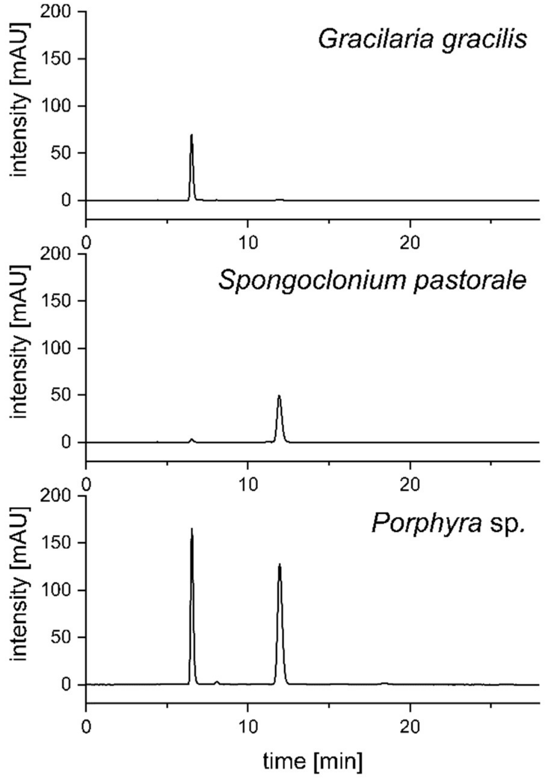


Figure S1. HPLC analysis of the crude algal extracts (2 mg/ml in water) monitored at 330 nm. HPLC conditions: YMC ODS C18 RS column (250 x 4.6 mm, 5 µm particle size); mobile phase: water with 20 mM ammonium formate and 0.25% formic acid (A) and methanol (B); 0% B from 0 to 20 min, to 20% B at 30 min, 98% B at 35 min, held for 5 min, re-equilibration 15 min; flow rate: 0.65 ml/min; column temperature: 8°C; injection volume: 5 µl.

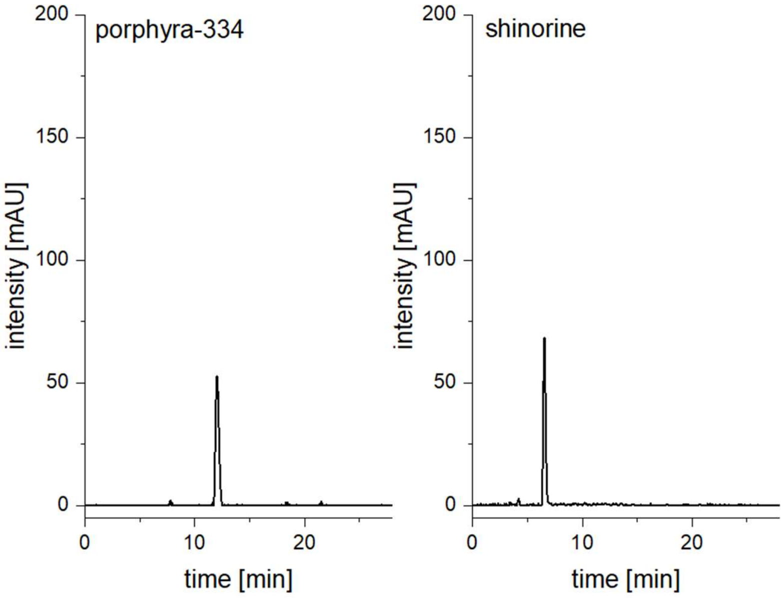


Figure S2. HPLC analysis of pure shinorine and porphyra-334 monitored at 210 nm; analytical conditions as above.

## 3. Online FCPC chromatograms

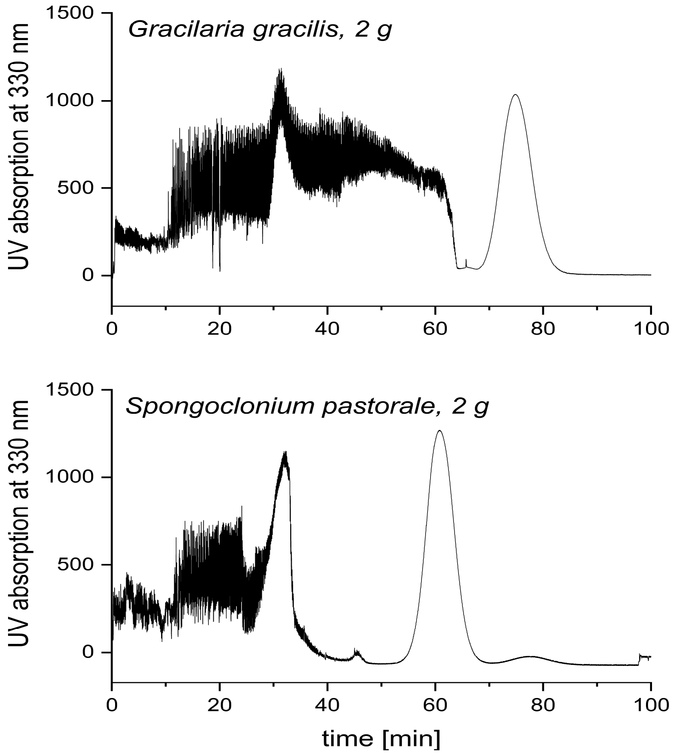


Figure S3. Online chromatograms recorded during the FCPC separation of the crude extracts of *G. gracilis* and *S. pastorale:* 2 g each in 30 ml biphasic system on the 1 l rotor; ATPS comprising 51.4 w% water, 28.0 w% ethanol (96 v%), 18.2 w% ammonium sulfate and 2.4 w% methanol in ascending mode; $S_f$ = 63%; 60 bar; collection volume 20 ml; detection at 330 nm; unstable baseline at the beginning of the separation due to stationary phase extrusion.

## 4. TLC Analysis

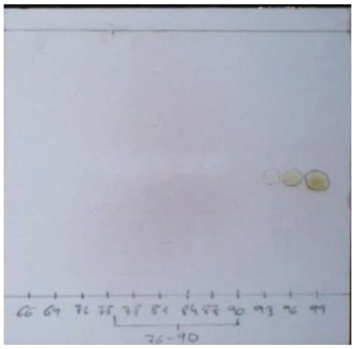

Figure S4. Removal of an impurity by fractionation exemplarily shown for *Porphyra* sp. TLC analysis of every third fraction during the elution of shinorine. A putative sugar (greenish band) eluted from fraction 93 onwards, the shinorine peak completed elution earlier (till fraction 90). The applied TLC solvent system was developed by Hartmann et al. and consisted of *n*-butanol: acetic acid: water 6:2:2 [7]. Anisaldehyde/sulphuric acid was used as spraying reagent, and the plates were derivatised at 100°C for 5 min. Evaluation of the TLC plates in VIS.

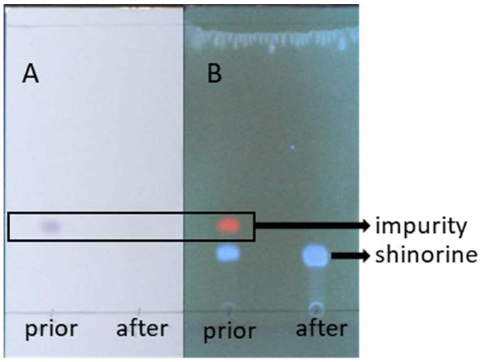


Figure S5. Purification of shinorine by Sephadex LH-20 column chromatography: **A**) TLC plate sprayed with Ninhydrin reagent before and after purification, VIS; **B**) TLC plate sprayed with Ninhydrin reagent before and after purification, 366 nm. TLC conditions as above.

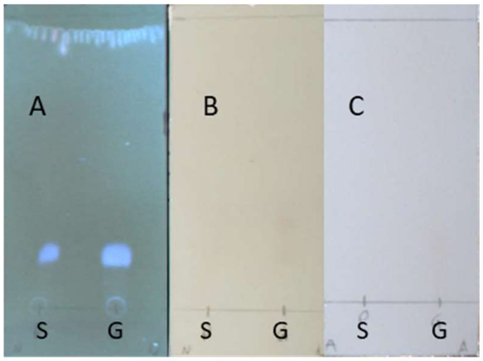


Figure S6. TLC of the pure compounds isolated from algae containing only one MAA: (S) *Spongoclonium pastorale* yielded porphyra-334; (G) *Gracilaria gracilis* yielded shinorine. “Empty” plates B and C are shown to confirm the absence of impurities (sugars, amino acids).

TLC-conditions as above

**A.** Spray reagent: ninhydrin, detection at 366 nm
**B.** Spray reagent: ninhydrin, detection in VIS
**C.** Spray reagent: anisaldehyde/$H_2SO_4$, detection in VIS

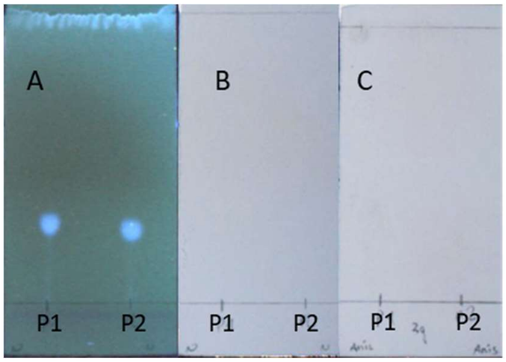


Figure S7. TLC of the pure MAAs isolated from 2g *Porphyra* sp. (Nori) extract: P1 corresponds to porphyra-334, P2 corresponds to shinorine. “Empty” plates B and C are shown to confirm the absence of impurities (sugars, amino acids).

TLC conditions as above

**A.** Spray reagent: ninhydrin, detection at 366 nm
**B.** Spray reagent: ninhydrin, detection in VIS
**C.** Spray reagent: anisaldehyde/$H_2SO_4$, detection in VIS

## 5. LC-MS Analyses

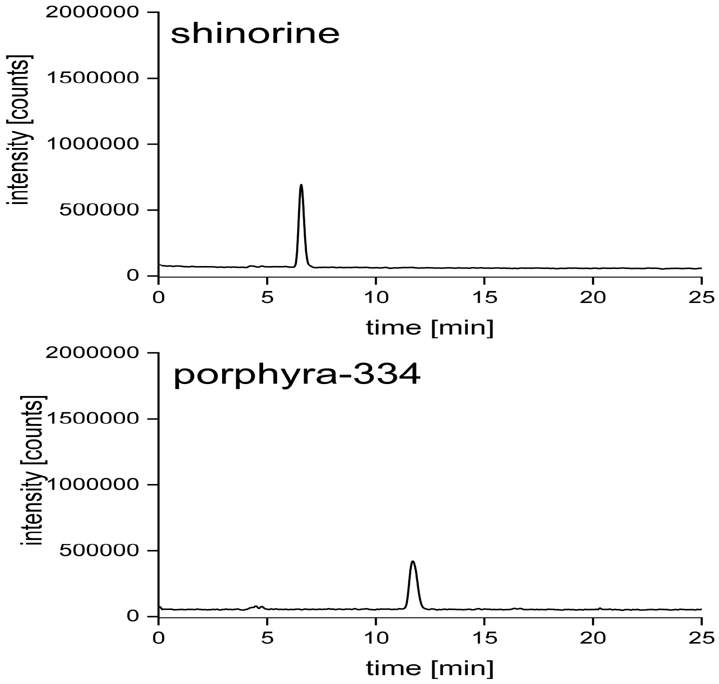


Figure S8. Total Ion Current (TIC) chromatogram of pure shinorine and porphyra-334. MS parameters: ESI negative mode; capillary voltage: 4500 V; drying gas: nitrogen at 12 l/min and 320 °C; nebulizer gas: nitrogen with 1.73 bar; scan range: 50 to 500 *m/z*. HPLC parameters: YMC ODS C18 RS column (250 x 4.6 mm, 5 µm particle size); mobile phase: water with 20 mM ammonium formate and 0.25% formic acid (A) and methanol (B); gradient: 0% B from 0 to 20 min, to 20% B at 30 min, 98% B at 35 min, held for 5 min, re-equilibration 15 min; flow rate: 0.65 ml/min; column temperature: 8°C; injection volume: 5 µl.

## 6. $^1H$ NMR spectra of isolated compounds

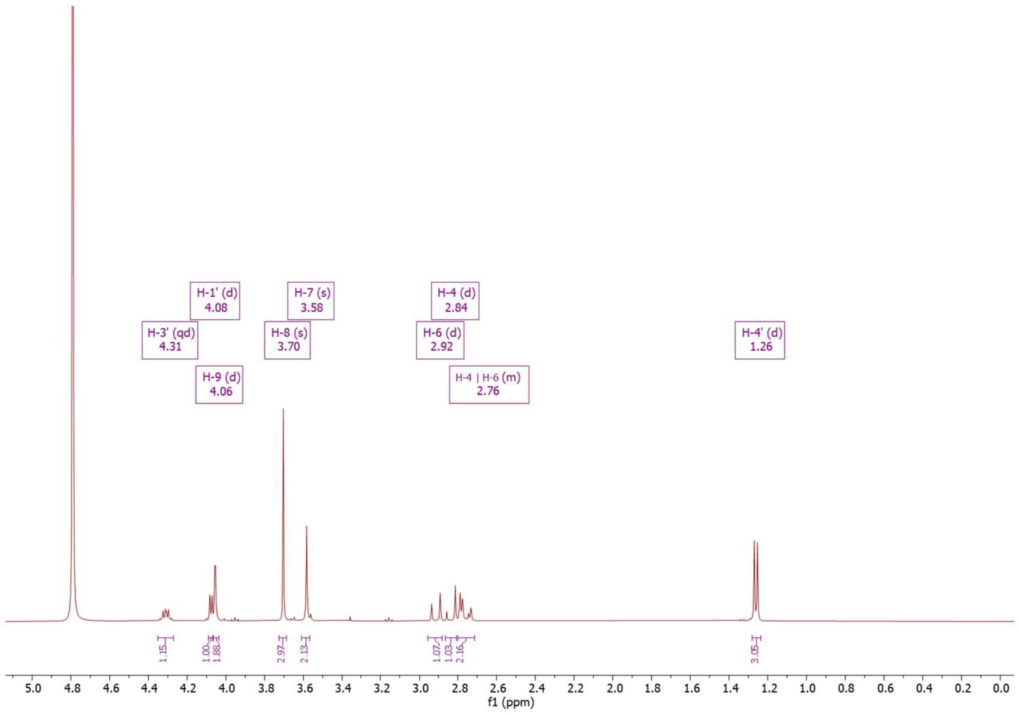


Figure S9. $^1H$ NMR spectrum of porphyra-334 (isolated from *Porphyra* sp.) recorded in $D_2O$ (400 MHz).

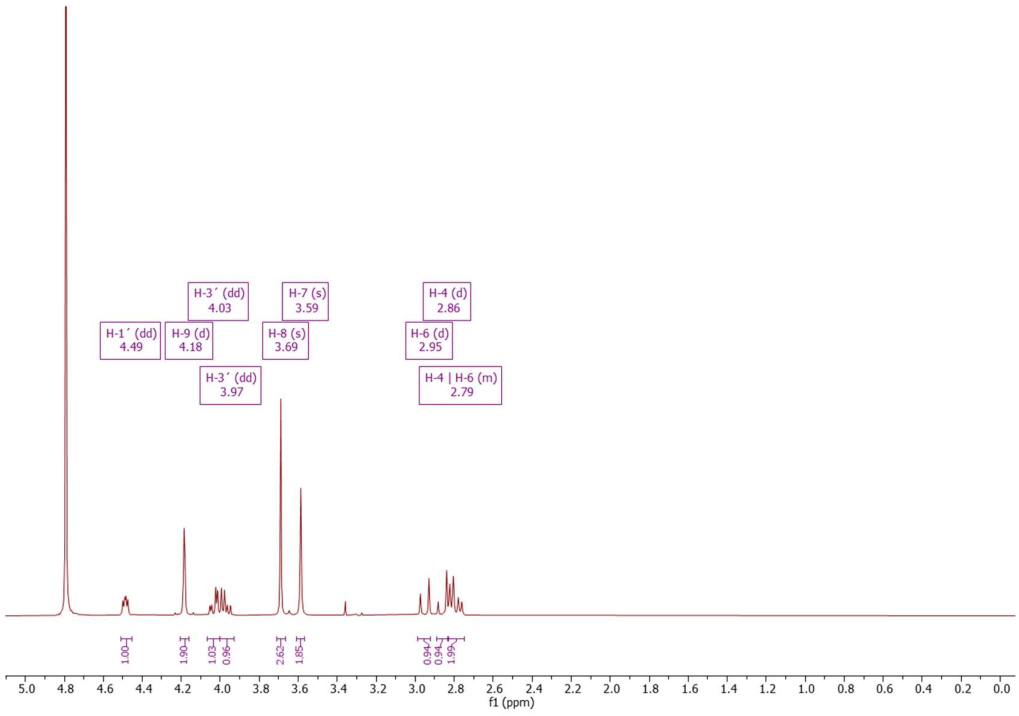


Figure S10. $^1H$ NMR spectrum of shinorine (isolated from *Prophyra* sp.) recorded in $D_2O$ (400 MHz).

Table S2. $^{1}$H-NMR data of isolated compounds in comparison to literature values; all spectra recorded in D2O at 400 MHz.

| **porphyra-334** | | **shinorine** | |
|---|---|---|---|
| **Literature values [1]** | **Found values** | **Literature values [1]** | **Found values** |
| **4.30 (1H, dq, J= 4.6, 6.4 Hz, H-3')** | 4.31 (1H, dq, J= 4.4, 6.5 Hz, H-3') | **4.48 (1H, dd, J=4.8, 6.4, H-1')** | 4.49 (1H, dd, J=3.9, 6.4, H-1') |
| **4.07 (1H, d, J=4.4 Hz, H-1')** | 4.08 (1H, d, J=4.6 Hz, H-1') | **4.18 (2H, d, J=3.1, H-9)** | 4.18 (2H, d, J=1.6, H-9) |
| **4.05 (2H, d, J=3.8 Hz, H-9)** | 4.06 (2H, d, J=1.7 Hz, H-9) | **4.02 (1H, dd, J=3.6, 12.0, H-3')** | 4.03 (1H, dd, J=3.8, 12.0, H-3') |
| **3.70 (3H, s, H-8)** | 3.70 (3H, s, H-8) | **3.96 (1H, dd, J=6.4,12.0, H-3')** | 3.97 (1H, dd, J=6.8,11.9, H-3') |
| **3.58 (2H, s, H-7)** | 3.58 (2H, s, H-7) | **3.67 (3H, s, H-8)** | 3.69 (3H, s, H-8) |
| **2.94 (1H, d, J=17.8 Hz, H-6)** | 2.92 (1H, d, J=17.5 Hz, H-6) | **3.57 (2H, s, H-7)** | 3.59 (2H, s, H-7) |
| **2.83 (1H, d, J=17.8 Hz, H4)** | 2.84 (1H, d, J=17.5 Hz, H-4) | **2.93 (1H, d, J=17.8, H-6)** | 2.95 (1H, d, J=17.5, H-6) |
| **2.77 (1H, d, J=17.8 Hz, H-4)** | 2.77 (2H, m, J=17.5, 17.1 Hz, H-4, H-6) | **2.84 (1H, d, J=17.8, H-4)** | 2.86 (1H, d, J=17.5, H-4) |
| **2.75 (1H, d, J=17.8 Hz, H-6)** | | **2.79 (1H, d, J=17.8, H-4)** | 2.79 (2H, m, J=17.4, 17.3, H-4, H-6) |
| **1.26 (3H, d, J=6.4 Hz, H-4')** | 1.27 (3H, d, J=6.5 Hz, H-4') | **2.79 (1H, d, J=17.8, H-6)** | |

[1] Orfanoudaki, M.; Hartmann, A.; Karsten, U.; Ganzera, M., Chemical profiling of mycosporin-like amino acids in twenty-three red algal species. *J Phycol* **2019**, 55, (2), 393-403.
